# Translation Levels Control Multi-Spanning Membrane Protein Expression

**DOI:** 10.1371/journal.pone.0035844

**Published:** 2012-04-26

**Authors:** Hok Seon Kim, James A. Ernst, Cecilia Brown, Jenny Bostrom, Germaine Fuh, Chingwei V. Lee, Arthur Huang, Richard L. Vandlen, Daniel G. Yansura

**Affiliations:** 1 Department of Antibody Engineering, Genentech Inc., South San Francisco, California, United States of America; 2 Department of Early Discovery Biochemistry, Genentech Inc., South San Francisco, California, United States of America; 3 Department of Protein Chemistry, Genentech Inc., South San Francisco, California, United States of America; Bernhard Nocht Institute for Tropical Medicine, Germany

## Abstract

Attempts to express eukaryotic multi-spanning membrane proteins at high-levels have been generally unsuccessful. In order to investigate the cause of this limitation and gain insight into the rate limiting processes involved, we have analyzed the effect of translation levels on the expression of several human membrane proteins in *Escherichia coli (E. coli)*. These results demonstrate that excessive translation initiation rates of membrane proteins cause a block in protein synthesis and ultimately prevent the high-level accumulation of these proteins. Moderate translation rates allow coupling of peptide synthesis and membrane targeting, resulting in a significant increase in protein expression and accumulation over time. The current study evaluates four membrane proteins, CD20 (4-transmembrane (TM) helixes), the G-protein coupled receptors (GPCRs, 7-TMs) RA1c and EG-VEGFR1, and Patched 1 (12-TMs), and demonstrates the critical role of translation initiation rates in the targeting, insertion and folding of integral membrane proteins in the *E. coli* membrane.

## Introduction

High-level expression of eukaryotic multi-spanning membrane proteins is particularly difficult in *E. coli* for unknown reasons. While many eukaryotic proteins can be secreted into the periplasm in significant quantities, it remains unknown what limits the accumulation of these polytopic membrane proteins.

Eukaryotic and prokaryotic cells share significant homology in both co-translational and post-translational membrane protein insertion mechanisms [1]. In prokaryotes such as *E. coli*, the post-translational mechanism is used primarily for secreted periplasmic proteins while a co-translational mechanism is used for integral membrane proteins [2]. However, in the cells of higher eukaryotes, such as mammals, the co-translational system is used almost exclusively for both integral membrane and secreted proteins.

Co-translational membrane insertion proceeds through several biochemical steps involving three different protein complexes. Initially, the signal recognition particle (SRP) recognizes and binds the first transmembrane or signal peptide domain as it emerges from the ribosome. A SRP receptor (SR) [1] binds to the SRP and docks the ribosome with the protein-conducting channel of the translocon, which creates a pore for insertion of the emerging polypeptide across the lipid bilayer. The hydrophobicity of a region of 20 to 40 residues in the emerging N-terminal domain of the nascent polypeptide determines the engagement of the SRP, and adjacent charged residues determine the orientation of the peptide in the cell membrane [3]. It has been shown that certain components of the *E. coli* SRP can be functionally substituted for their eukaryotic homologues [4], emphasizing the similarities of the two systems.

The number of SRP complexes in eukaryotes suggests one important difference in protein membrane targeting mechanisms. Eukaryotic cells typically contain approximately 10,000 copies of SRP particles or approximately 1 SRP per 10 ribosomes [5]. By comparison, the prokaryotic SRP is present at much lower copy number, often just a single SRP per 100 to 1,000 ribosomes, or as few as 50 particles per cell.

The eukaryotic and prokaryotic SRP also have different regulatory functions. In *E. coli*, the Ffh-4.5S RNA component of the SRP does not contain a functionally analogous region to the *Alu* domain of the eukaryotic SRP [6], [7] and thus lacks a corresponding translation pause mechanism.

Further compounding the regulatory differences between eukaryotes and prokaryotes, translation elongation rates in *E. coli* cells can exceed the rate in eukaryotic cells by as much as ten fold. All of these factors result in an extremely short time period during which the emerging hydrophobic polypeptide chain in *E. coli* may interact effectively with the membrane bound translocation machinery, unless some other pause mechanism exists.

Several mechanisms have been postulated to explain the problems with membrane protein expression. These rationales include available membrane area and protein crowding in the membrane space, general transmembrane protein toxicity [8] and stability of the protein sequence itself [9]. Since the area of plasma membrane per volume in a eukaryotic cell is smaller than the area of plasma membrane per volume in a prokaryotic cell, simply based on cell size, it is unlikely that the amount of membrane is a limiting factor in protein expression. Likewise, since several proteins, the KcsA potassium channel [10], and bacteriorhodopsin [11], among others, can be expressed at several milligrams per gram of cell mass, it is unlikely that protein crowding in the plasma membrane is a limiting factor in expression. Previous attempts to improve membrane protein expression in *E. coli* have relied on selective screening to identify random mutations in specific bacterial strains [12], [13]. With few exceptions, improvements were limited to bacterial proteins and rarely resulted in increased expression per cell. Attempts to address expression problems with simple N or C terminal tags have had limited success [14] while evaluation of various promoter systems has also shown similar modest improvement.

Our study focused on determining the influence translation levels have on the expression of eukaryotic multi-spanning membrane proteins in *E. coli*. Using different leaders to control translation initiation, we show that translation initiation rates determine both initial induction rates and total protein accumulation. High translation rates quickly lead to a halt in synthesis while more moderate rates allow for high levels of accumulation over an extended period of time.

## Results

### Expression of membrane proteins

**Figure 1:**
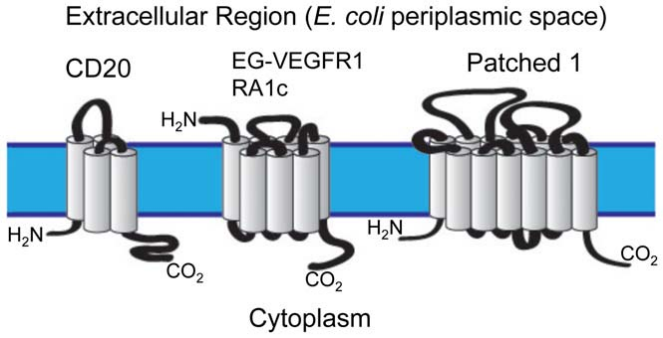
Topology diagram of model human proteins as expressed in a mammalian cellular plasma membrane. The predicted molecular weights for these proteins without post-translational modification are: CD20, 33.0 kDa; EG-VEGFR1, 44.8 kDa; RA1c, 35.5 kDa and Patched 1, 160.5 kDa.

Earlier work assessed the expression of CD20, a marker of human B-cells with four alpha-helical membrane domains [15]. That study demonstrated isolation of milligram quantities of CD20 in a native like conformation from the bacterial membrane, following expression from a vector previously described for *E. coli* protein production. In the current study, we attempted to extend this work to three new candidate proteins: the human G protein coupled receptors (GPCRs), RA1c [16], [17] and EG-VEGFR1 [18], [19], [20] with 7-TM domains, and the 12-TM transport like protein Patched 1 [21], [22]. Topology diagrams and molecular weights of the candidate proteins in their native state are shown in figure 1. These proteins were chosen solely based on their biological roles or potential as therapeutic targets.

**Figure 2:**
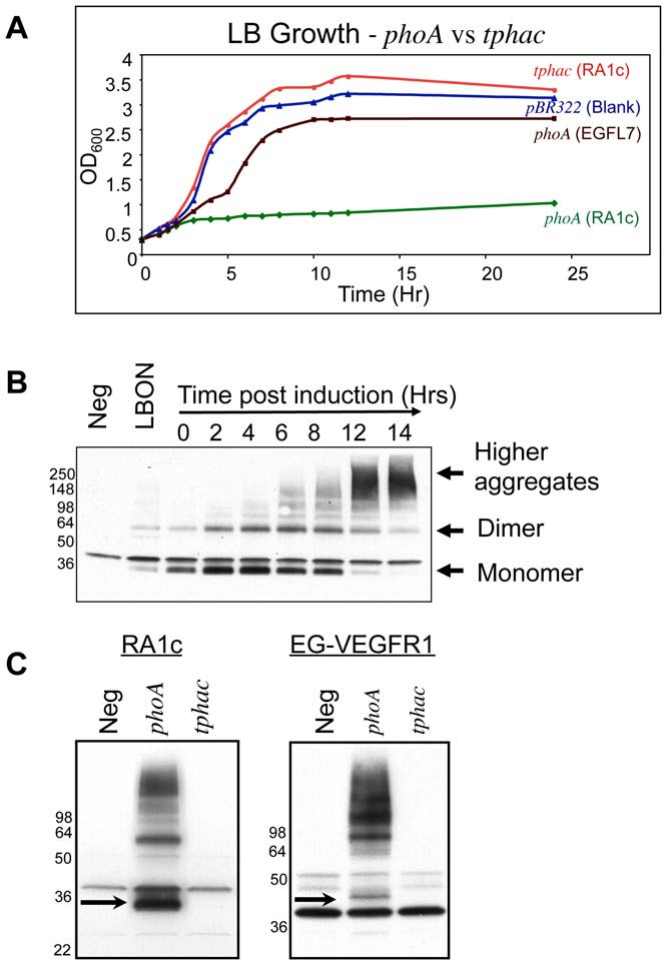
Improved cell growth and general accumulation of integral membrane proteins using a dually regulated promoter. (**A**) Restricted *E. coli* growth in LB with the *phoA*-RA1c construct is relieved by using the *tphac* promoter, which reduces basal level expression. A 24-hour growth curve shows the empty pBR322 vector control (blue triangles), *phoA*-RA1c expression construct (green diamonds), *tphac*-RA1c expression construct (red circles) and *phoA*-EGFL7 as a non-membrane protein control (brown squares). (**B**) A representative western blot of RA1c expression from the *phoA* promoter is shown following induction by phosphate depletion when the cells reach approximately 2 OD_600_ (time 0). Maximum expression is reached within two hours post induction. By 6 hours, aggregation has begun and by twelve hours almost all the protein has moved from the monomer band to high molecular weight aggregate. Basal expression is shown after overnight growth in LB medium (LBON). The western blot was probed with an HRP coupled anti-his antibody. (**C**) A comparison of basal expression in LB of the GPCR proteins, RA1c and EG-VEGFR1, from the *phoA* and *tphac* promoters by western blot analysis. The *phoA* constructs show significant accumulation levels of the membrane proteins while the *tphac* constructs have reduced the accumulation to background levels. The arrow points to the monomer protein band.

The three candidate genes were inserted into the original expression vector under the transcriptional control of the *phoA* promoter. In addition, each gene contains nucleotide sequences encoding a small seven amino acid MKHQHQQ (Uni) leader to provide an efficient translation initiation. Induction of CD20 and Patched 1 by means of phosphate limitation resulted in a stable level of protein expression over time, detectable by anti-his western (data not shown). However, both GPCR constructs had a significant toxic effect on the host as demonstrated by the size of the bacterial colonies (Figure S1). In addition, expression of either GPCR was problematic and variable due to the poor growth in even transcription-repressed conditions (Figure 2A). Further, we observed a striking pattern in the expression time course for both proteins. Both monomer and dimer forms of the two GPCRs were stable for about four hours post induction, after which time the proteins were transformed into high molecular weight aggregates. This can be clearly seen for RA1c in figure 2B. The time course of this transition suggests the proteins are correctly membrane inserted initially, but become highly aggregated over time.

### Basal level transcription controlling

Toxicity from basal expression of the GPCR constructs in *E. coli* created significant experimental variability, which complicated any controlled study. To reduce basal transcription, we inserted the *lac* operator [23], [24] at the +1 position of the existing *phoA* promoter [25], [26]. The resulting *phac* promoter requires both phosphate starvation as well as the addition of the *lac* inducer, isopropyl β-D-1-thiogalactopyranoside (IPTG) for full induction (Figure S2). Partial induction levels can be achieved by manipulating each regulatory element individually in a *lac* repressor *iQ* strain. To further suppress possible upstream cryptic promoters, the λ t_o_ transcriptional terminator [27] was introduced upstream of the *phac* to create the *tphac* promoter. Subsequent work has shown that the *phac* and the *tphac* promoters behave similarly.

The *phoA* promoter in the GPCR expression constructs was replaced with the *tphac* promoter to assess whether or not basal level toxicity was still a problem. A comparison of the colony sizes of the *E. coli* host after transformation with the EG-VEGFR1 and RA1c plasmids suggested that the *tphac* promoter had significantly reduced the toxicity of both genes (shown for RA1c in Figure S1). The subsequent culture of these colonies in non-inducing conditions showed that there was no growth retardation with the *tphac* promoter as compared to those constructs with the *phoA* promoter (Figure 2A). Finally, basal accumulation of the GPCRs with the *phoA* promoter was reduced to background with the *tphac* promoter (Figure 2C).

### Alteration of translation levels

To gain insights into the effects translation levels have on membrane protein expression, we initially attempted to increase translation levels to see if these membrane proteins could be forced into refractile bodies. As the Uni leader is optimized for translation initiation within the constraints of its coding sequence, we incorporated a new leader, which had previously been shown to result in exceptionally high translation levels, the *trp* LE. The *trp* LE was originally isolated as a fusion of the first 9 amino acids of the *trp* leader to distal parts of the E protein encoded in the *trp* operon [28]. We designed a leader based on the first 79 amino acids of the LE and fused this to the N-termini of each of the four studied proteins.

**Figure 3:**
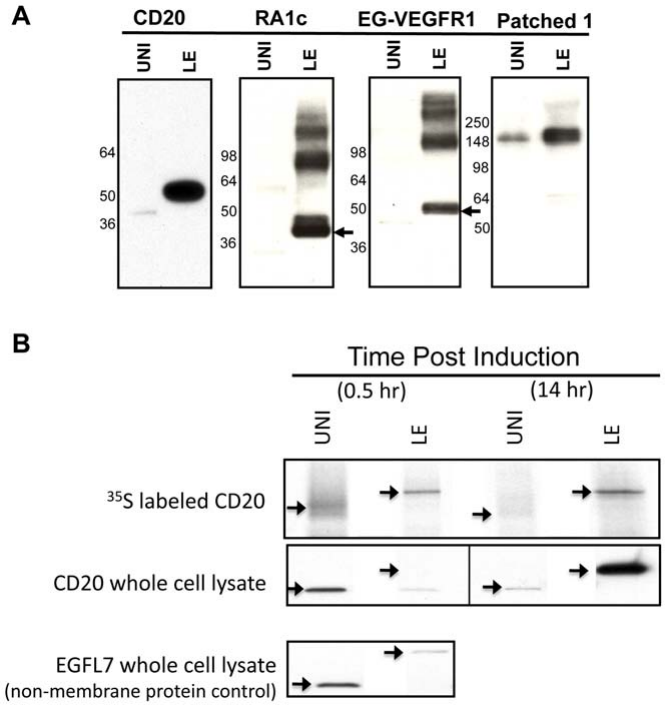
Improved integral membrane protein expression with the LE leader. (**A**) Comparison of the expression levels with the Uni and the LE leaders for multi-spanning membrane proteins CD20, RA1c, EG-VEGFR1 and Patched 1. Arrows point to the monomer protein bands for the two GPCRs. (**B**) The Uni leader has a higher translation rate than the LE leader at the beginning of the induction, but the rates reverse by the end of the induction. Relative translation rates were measured by pulse labeling cells expressing CD20 with ^35^S cysteine for five minutes as well as by assessing accumulation levels in whole-cell extracts by immunoblot with HRP conjugated anti-His antibody. The non-membrane protein EGFL7 was used as a control.

Production of the four membrane proteins with the LE leader was compared to the original constructs using the smaller Uni leader. The *tphac* promoter was induced as noted earlier and a comparison of the leaders was made at 12 hours post IPTG addition. All four proteins with the LE leader showed a significant increase in production as shown in figure 3A. The increase in the accumulation of the two GPCRs was particularly striking and higher than expected for a modest change in translation initiation rates. Expression levels of these membrane proteins with the Uni leader were also compared to constructs with no leader. The lack of a leader resulted in very low expression compared to the Uni with CD20, RA1c and EG-VEGFR1, while Patched 1 expression was modestly higher than that observed with the Uni leader (Figure S3). However, because the translation initiation rates of the constructs without leaders are unknown, it is difficult to interpret these results.

Control experiments fusing the native Met Patched 1 translation initiation region (TIR- first seven residues) to an unrelated protein EGFL7 show that the native Patched 1 TIR is extremely weak, and no translated protein could be detected in experiments similar to those shown in figure 3B (data not shown). The first 160 base pairs at the start of the Patched 1 gene are highly G/C rich (79%), and significant mRNA secondary structure would be expected to inhibit translation initiation at the planned start for the Met construct, and possibly the Uni construct. The most likely explanation for the minor expression observed from the native Met Patched 1 TIR in figure S3 is internal translation from Met152, which possesses a good Shine-Dalgarno just upstream. Translation from this residue is consistent with the observed molecular weight.

### Comparison relative translation rates

In order to compare the relative translation initiation rates for the two leaders, CD20 synthesis rates were determined early in the induction. Cells were induced for 30 minutes after which samples were removed for western blot analysis of CD20 accumulation with each leader. The culture was then labeled with ^35^S cysteine for 5 minutes, and his-tagged CD20 was isolated by Ni-NTA resin. After separation by SDS-PAGE and transfer to nitrocellulose, CD20 was visualized either by anti-his western blot or autoradiography. Surprisingly, the results shown in figure 3B reveal that the Uni leader has a higher translation rate than the LE leader early in the induction. A similar experiment with the two leaders was performed with the non-membrane His-tagged EGFL7, a protein which aggregates in the cytoplasm. Again, the Uni leader reveals a stronger translation rate than the LE leader (Figure 3B). However, if the CD20 culture is pulse-labeled for 5 minutes with ^35^S cysteine later in the induction (14 hours post IPTG addition), then the translation rate for the LE leader is much higher than that observed for the Uni leader (Figure 3B).

**Figure 4:**
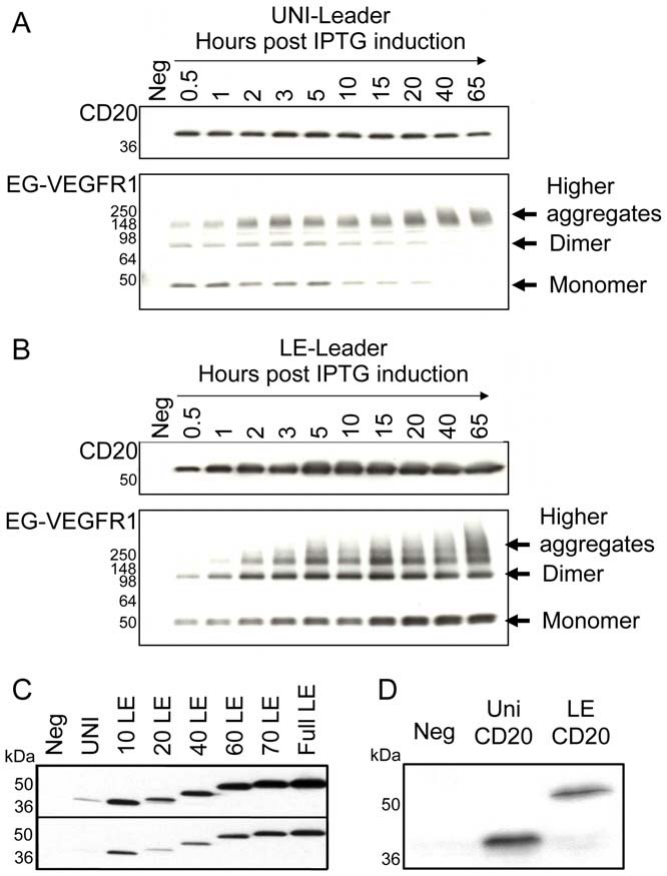
Leader dependent accumulation of CD20 and EG-VEGFR1 following induction of the *tphac* promoter. (**A**) Protein accumulation maximizes within thirty minutes of induction with the Uni leader. (**B**) Protein accumulation continues over 20 hours after induction with the LE leader. (**C**) The effect of C-terminal truncations on expression from the LE leader. The full 79 amino acid LE leader was truncated from the C-terminus to observe the importance of the translation initiation rate as compared to the length of the LE leader. Truncated leaders were fused to CD20 and the whole cell lysates were immunoblotted with HRP conjugated anti-His antibody. Two film exposures are shown. (**D**) Reduced promoter activity results in reversal of the relative expression levels from the Uni and the LE leaders fused to CD20. Cultures were grown under partial promoter induction and the whole cell lysates were probed with HRP conjugated anti-His antibody for detection.

Immediately following induction, translation from the Uni leader is higher than from the LE leader; however, the relative rates of the two leaders reverse over time. To examine this observation in more detail, the induction of CD20 or EG-VEGFR1 fused to each of the two leaders was repeated and samples were removed at numerous time points. These samples were analyzed by SDS-PAGE followed by anti-His western blotting to visualize CD20 and EG-VEGFR1 accumulation. As shown in figure 4A and B, the accumulation of both CD20 and EG-VEGFR1 reaches a maximum after approximately 30 minutes with the Uni leader. By contrast, accumulation of both membrane proteins from the LE leader increased over several hours to outpace accumulation from the Uni leader.

### Leader amino acid sequence and size are not important

To confirm that the translation initiation rate is the crucial variable in expression of these membrane proteins, the length of the LE leader was evaluated for effects on protein accumulation. A series of deletions at the C-terminus of the LE leader were created while preserving the TIR [29] in the first several codons. These constructs were fused to the N-terminus of CD20 and analyzed for their ability to accumulate protein. The results shown in figure 4C reveal that CD20 accumulation does modestly and gradually decrease with size. However, even at the smallest size of 10 amino acids, the accumulation of CD20 with the LE leader is significantly greater than that of the Uni leader (7 amino acids). This suggests that the core TIR of the leader is important for membrane protein accumulation presumably as a function of translational strength.

The results imply that the weaker TIR of the LE leader allows continuous membrane protein accumulation over several hours of induction while the stronger TIR of the Uni leader produces an early overload and collapse of the membrane targeting system. Therefore, if transcription levels were equivalently reduced for both leaders, then overload of the membrane targeting system would be avoided and the Uni leader should surpass the LE leader in membrane protein accumulation. To test this hypothesis, cultures with either the Uni or the LE leader fused to CD20 under the control of the *tphac* promoter were induced by phosphate starvation for 16 hours. Without the addition of IPTG to remove the lac repressor control, a partial induction is achieved, leading to an equivalent drop in transcription/translation in each cell. The results shown in figure 4D bear out this prediction with the Uni leader, resulting in greater membrane protein accumulation than that observed with the LE leader and rule out anything special about their actual amino acid sequences.

### Membrane association of over-expressed proteins

**Figure 5:**
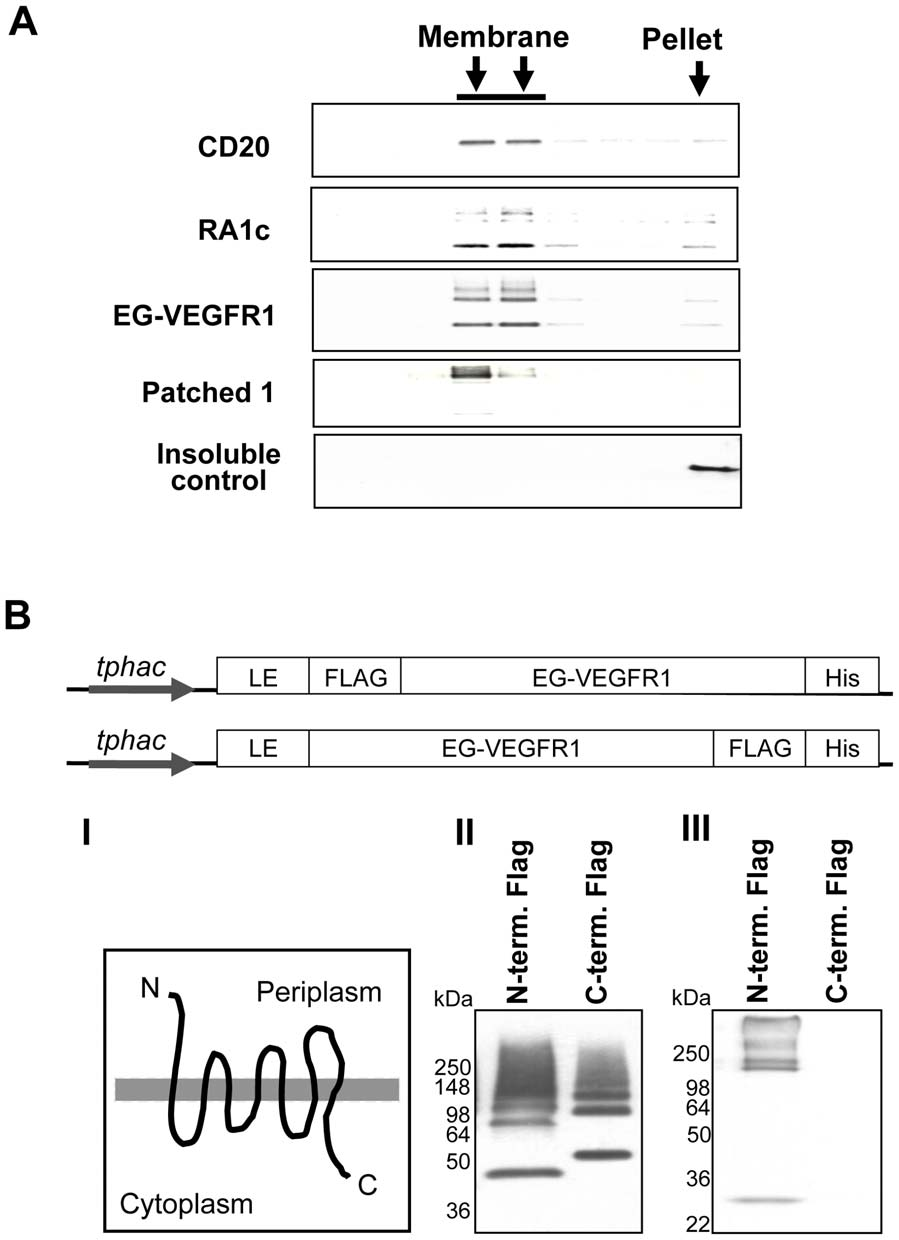
Membrane localization, cell surface expression and orientation of integral membrane proteins expressed with the LE leader. (**A**) Over-expressed LE tagged proteins are localized to the membrane fraction in sucrose density gradient centrifugation. The insoluble non-membrane protein EGFL7 has been included as a control. Each lane represents fractions removed from the sucrose gradient and analyzed by SDS-PAGE anti-his immunoblot. (**B**) Orientation in the *E. coli* membrane was established by creating two constructs of EG-VEGFR1 with a FLAG-tag epitope at either the N or C terminus. {I} GPCR transmembrane proteins are known to have an exposed N-terminus on the surface of cells. {II} EG-VEGFR1 constructs containing the FLAG-tag epitope at either the N or C terminus express at similar levels in *E. coli* as confirmed by anti-his immunoblot of whole cell lysates. {III} Only spheroplasts of cells expressing the FLAG tag epitope at the exposed N-terminus were able to pull-down anti-FLAG antibody as detected by anti-murine IgG HRP conjugated antibodies.

To ascertain the native like expression of proteins fused to the LE leader, the sub-cellular localization of the proteins was evaluated by equilibrium ultracentrifugation as previously described [15]. Correctly localized membrane proteins should migrate with the bacterial membranes to a density of less than 1.29 g/cm^3^ (1.75 M sucrose layer), while typical soluble or retractile body proteins, if present, lack membrane association and have a density between 1.33–1.42 g/cm^3^ and will migrate to the bottom of the sucrose gradient. The results shown in figure 5A indicate that all four LE tagged proteins migrate to above the 1.29 g/cm^3^ density layer, consistent with the 1.15–1.25 g/cm^3^ density of the *E. coli* membrane [30].

### Correct membrane orientation

To determine if the GPCRs with the LE leader are correctly oriented in the cytoplasmic membrane, a FLAG tag was added to either the N- or C- termini of EG-VEGFR1 and the extracellular localization of the FLAG tag was evaluated by immuno-precipitation of *E. coli* spheroplasts expressing either constructs [31]. These results shown in figure 5B-II demonstrate equivalent expression of both constructs. The FLAG antibody binds to LE-EG-VEGFR1 only when the FLAG tag is expressed at the N-terminus as predicted for a GPCR; however, no binding is observed for LE-EG-VEGFR1 with the FLAG tag at the C-terminus (Figure 5B-III). This indicates the FLAG tag is unavailable for binding, which is consistent with the cytoplasmic localization of EG-VEGFR1 C-terminus (Figure 5B-I). To confirm that the FLAG tag on LE-EG-VEGFR1 is equally accessible to antibody when placed at either the N or C terminus, we prepared *E. coli* membrane proteoliposomes where both sides of the membrane are accessible. In each case, approximately equivalent amounts of FLAG antibody were recovered by immuno-precipitation (Figure S4) Addition of the detergent Triton X-100 did not further enhance accessibility of the FLAG tag, as might be expected.

### Cell membrane expression of CD20

**Figure 6:**
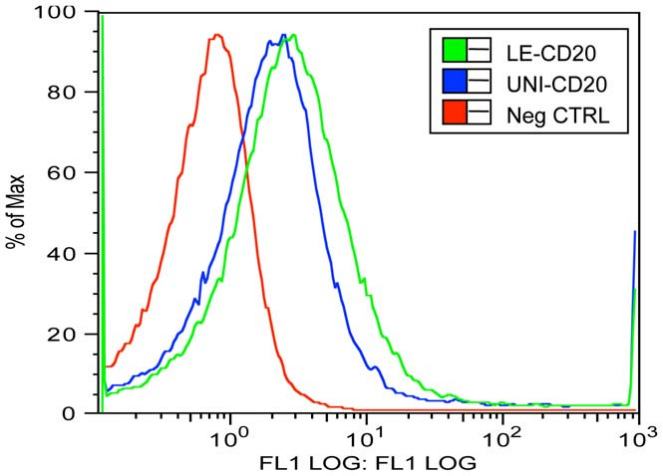
Expression and folding of CD20 in the *E. coli* inner membrane. Cell surface expression and orientation of CD20 was assessed from spheroplasts of *E. coli* cells expressing either Uni-CD20 (blue), LE-CD20 (green) or an empty control vector (red) treated with Alexa-488 conjugated anti-CD20 antibody to the extracellular loop of CD20 and analyzed by flow cytometry.

To confirm native folding and membrane expression of human CD20 in a cellular context, spheroplasts of *E. coli* expressing CD20 were evaluated by fluorescent activated cell sorting (FACS) [32] using the conformation dependent antibody rituximab [33]. The second of the two extracellular loops of CD20 is the binding site for rituximab and this interaction is strongly dependent on the native conformation stabilized by a disulfide bond. FACS analysis showed a large shift in mean fluorescent intensity for CD20 expressing cells as compared to control cells shown in figure 6. The data is consistent with localization of CD20 to the cytoplasmic membrane and correct native like folding of the second exracellular loop of CD20.

### High-expression yields

**Figure 7:**
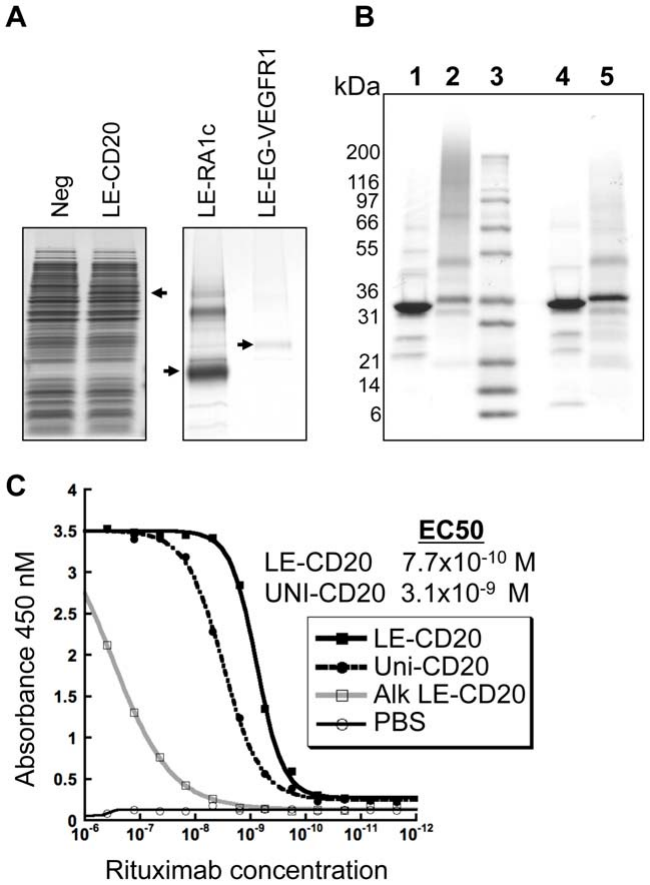
Protein expression, isolation and characterization. (**A**) CD20 (left most panel) could be seen in whole cell extracts, while RA1c and EG-VEGFR1 (right panel) required purification on Ni-NTA resin. (**B**) Coomassie-stained SDS gel of purified CD20. Lane 1) LE-CD20 non-reduced; 2) Uni-CD20 non-reduced; 3) Molecular weight markers: 200, 116, 97, 66, 55, 36.5, 31, 21.5 14.4 6 kDa; 4) LE-CD20 reduced; 5) Uni-CD20 reduced. (**C**) Activity of Uni and LE human CD20. Activity of the isolated proteins was assayed using the conformation specific antibody rituximab. Binding to LE-CD20 (solid black line, solid squares), Uni-CD20 (dashed line, solid circles), reduced and alkylated LE-CD20 (negative control) (solid gray line, open squares), and (control) PBS (solid black line, open circles). The curves for rituximab binding were determined from a 4-parameter fit and the LE leader was cleaved from CD20 before analysis.

Extraction of proteins using native detergents confirmed the high expression levels with the LE leader and the *phac* promoter. Western blots suggest that approximately 90% of CD20, RA1c or EG-VEGFR1 are extracted in Fos-Choline 12 (FC12); however, Patched 1 is largely resistant to extraction in this detergent. In addition, LE-CD20 can be extracted in a mixture of FC12 and dodecyl maltoside (DDM) detergents (Figure S5) further indicating a native like conformation of this protein in the membrane [34]. The detergent FC12 has demonstrated excellent properties for solubilizing the *E. coli* membrane [35], and Fos-Choline detergents and FC12 have shown favorable properties for the isolation of eukaryotic membrane proteins [36] including GPCRs [37]. Expression of LE-CD20 can be detected in coomassie stained whole cell extract, while the GPCR proteins require additional enrichment using Ni-NTA resin (Figure 7A). Single step IMAC purification of all three proteins provide 2 to 10 mg of protein per liter at greater than 90% purity as estimated from coomassie stained gels (Figure S6). Large and small-scale isolations of CD20, RA1c, and EG-VEGFR1 (Table S1) show yields are reproducible within two fold. Quantification of LE-CD20 expression levels in whole cell extracts against a standard curve of purified LE-CD20 show total cellular expression levels to be 41 mg/L (Figure S7 and Methods S1), indicating 25% protein recovery after primary purification. We estimate recovery of EG-VEGFR1 and RA1c to be similar. The high LE-CD20 expression levels translate to 3×10^5^ molecules per cell – consistent with FACS data.

### Characterization of LE-CD20

Earlier functional expression and purification of CD20 demonstrated isolation of 10–20 µg of His-tagged protein from a gram of *E. coli* cells. For comparison, LE tagged human CD20, under the transcriptional control of the *tphac* promoter, was expressed in *E. coli* and isolated from cell membranes by IMAC affinity chromatography followed by thrombin cleavage of the LE leader and size exclusion chromatography. Representative samples of purified his-tagged human CD20 are shown in the SDS polyacrylamide gel in figure 7B. CD20 isolated in this relatively simple manner is over 95% pure with a final yield better than 5 mg/L of protein in shake-flasks or 1 mg/g cells. The protein migrates with an apparent molecular weight of approximately 35 kDa under reducing conditions, which is in reasonable agreement with the calculated molecular weight of 33 kDa. In both reducing and non-reducing SDS-PAGE, purified LE-CD20 shows significantly fewer contaminating protein bands and less protein aggregate than Uni-CD20, consistent with the improved expression properties of LE-CD20.

To confirm proper folding and processing of LE-CD20, the presence of the disulfide bond in the extracellular domain of CD20 was evaluated using the conformation specific antibody rituxmab [33] in the ELISA assay described previously [15]. In this assay, rituximab binds LE-CD20 with an EC_50_ of 0.77 nM (Figure 7C). This affinity is tighter than the binding of rituximab to control Uni-CD20 of 3.1 nM and in reasonable agreement with previously reported data [15]. As an additional control, LE-CD20 was reduced and alkylated and assayed for rituximab binding. This procedure eliminates rituximab binding, thus confirming the proper formation of the CD20 extracellular disulfide bond.

### Ligand binding to LE-EG-VEGFR1

**Figure 8:**
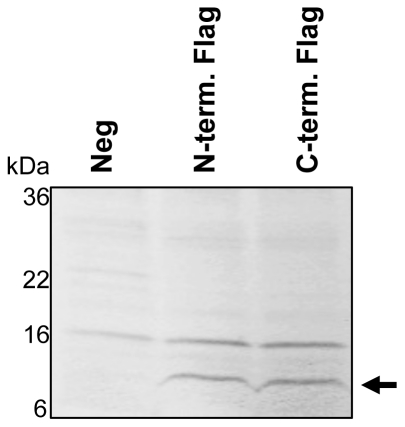
Ligand binding to the GPCR, LE-EG-VEGFR1. *E. coli* membrane proteoliposomes were treated with thrombin to remove the LE-leader and incubated overnight at 4°C with EG-VEGF in PBS. Pelleted membranes were separated by SDS-PAGE and developed by immuno-blot using an anti-EG VEGF antibody. Samples are: lane 1) pBR322 negative control; 2) LE-EG-VEGFR1, N-terminal FLAG; 3) LE-EG-VEGFR1, C-terminal FLAG. The location of EG-VEGF (molecular weight 9 kDa) is indicated by an arrow.

To demonstrate proper folding and function of one of the GPCRs, we evaluated ligand binding to LE-EG-VEGFR1 (RA1c has no known ligand). EG-VEGF (Prokineticin 1) was incubated with *E. coli* membrane proteoliposomes prepared from negative control cells and cells expressing LE-EG-VEGFR1 fused to a FLAG epitope at either the N or the C terminus. These membranes were extensively washed, pelleted by centrifugation and analyzed by SDS-PAGE and developed by immuno-blot using an antibody to EG-VEGF. As shown in figure 8, the EG-VEGF ligand binds to LE-EG-VEGFR1 membrane proteoliposomes, indicating at least some population of the receptor is properly folded.

Although experimental conditions limit exact quantitation of the amount of ligand bound to the receptor, we estimate the amount of receptor bound EG-VEGF in these experiments at 2×10^3^ molecules/cell, from a series of known concentrations of the ligand. Based on our results for receptor expression and recovery (Table S1 and Figure S7), we estimate the receptor at approximately 9×10^3^–4×10^4^ molecules/cell. Accounting for the loss of correct orientation of the receptor in the membrane following generation of proteoliposomes, we estimate that 10–40% of the receptors are able to bind ligand.

## Discussion

The expression of eukaryotic multi-spanning membrane proteins in *E. coli* is particularly difficult compared to the relative ease of producing cytoplasmic and secreted proteins. A number of efforts have been undertaken in different labs to identify and overcome the expression barrier with this class of proteins. This work includes the use of special bacterial strains [12], reduced transcription [13], proteomic analysis upon induction [8] and a variety of different affinity tags [14]. However, with the exception of a recent report involving the directed evolution of a GPCR that resulted in greater expression and stability [9], accumulation of these proteins per cell remained about the same. Additionally, the underlying molecular limitation of expression has remained elusive.

Our study focused on the relationship between translation levels and the expression or accumulation of these membrane proteins in *E. coli*. Since translation levels are largely determined by the translation initiation rate, we began by comparing the expression of four mammalian multi-spanning membrane proteins fused to two previously described leaders, the Uni and the LE. The resulting expression per cell of membrane proteins with the two leaders varied by 1–2 orders of magnitude – a much larger than expected difference considering that both leaders were thought to have similar high translation rates. Subsequent analysis revealed that the LE leader, which produced much higher levels of membrane protein expression, actually had a translation initiation rate that was several fold lower than that of the Uni leader.

A detailed look at the expression profile shows that the Uni leader with its stronger translation rate does indeed outpace the weaker translating LE leader very early in the induction. However, after approximately 30 minutes, membrane protein expression with the Uni leader slowed significantly with no further increase in protein accumulation. By contrast, the LE leader membrane protein expression and accumulation continued without change for several hours. This allowed the more slowly translating LE leader membrane proteins far greater total production than the initially highly translating Uni leader membrane protein.

All four highly expressed mammalian proteins with the LE leader are membrane associated upon cell fractionation, and inserted with a native like structure in the cell membrane. We analyzed the orientation of one of the GPCRs, EG-VEGFR1. The N-terminus of this receptor is orientated towards the periplasm while the C-terminus is cytoplasmic as is expected for proper insertion. The receptor also shows binding to its ligand, EG-VEGF. Additionally, CD20 has the correct orientation in the membrane based on FACS analysis and rituximab antibody binding.

The effect of translation levels on multi-spanning membrane protein expression can be quite significant and this needs to be understood at the molecular level. The most likely explanation for our observations is a potential bottleneck at the level of membrane targeting, presumably by the SRP. Translation at too high of a rate would be expected to overload the more limited co-translational secretory pathway in *E. coli* and quickly lead to a halt in translation as we observe with the Uni leader. A more moderate level of translation seen with the LE leader avoids an overload of this pathway, allowing for membrane targeting and insertion over longer periods of time. The halt in translation observed with the Uni leader soon after promoter induction suggests that there is a mechanistic membrane protein targeting-translation coupling that is maintained in the cell, although, the exact molecular nature of this coupling remains to be determined.

Optimizing integral membrane protein accumulation could potentially be controlled at the level of transcription to achieve the desired translation rate. The promoter sequence could be modified to provide a specific transcriptional strength, or alternatively repressor controlled promoters could be induced with suboptimal inducer concentrations. This later approach, however, requires the deletion of inducer transporters [38] and limits host strain selection.

## Materials and Methods

All *E. coli* experiments used the 58F3 strain which was derived from W3110 and has the following genotype *Δfhu (ΔtonA) Δlon galE rpoHt (htpRts) ΔclpP lacIq ΔompTD(nmpc-fepE) ΔslyD*.

All detergents were obtained from Anatrace, Inc., Maumee, OH. Unless otherwise mentioned, all chemicals were obtained from Sigma-Aldrich, St. Louis, MO.

Rituximab antibody was obtained from Genentech Manufacturing, anti-His from Roche, anti-FLAG M2 from Sigma and anti-Prokineticin 1 from Novus Biologicals.

### Cloning and Expression

The cDNA for human CD20, RA1c, EG-VEGFR1, and patched 1 were sub-cloned, using standard molecular biology techniques, into a pBR322-derived plasmid containing the β-lactamase gene and tRNA genes for three rare *E. coli* codons (*argU*, *glyT* and *pro2*). A short Uni (MKHQHQQ) and 79 amino acid LE sequence were added to the N-terminus of the membrane proteins and an octa-His sequence was placed at the C-terminus to aid in detection and purification. A thrombin cleavage site (LVPRGS) has been placed after the LE leader to allow removal of the leader sequence. Gene transcription is under control of the *phoA*, *phac* or *tphac* promoter. Gene expression was induced by dilution of a saturated LB carbenicillin culture into C.R.A.P. phosphate limiting media [39]. The culture was then grown at 30°C for 24 hours or the specified time for the *phoA* promoter. The Pho regulon generally turns on approximately 7–8 hours post dilution when the cell density reaches an optical density at 600 nm (OD_600_) of 2. For the *tphac* promoter induction, cultures were induced at OD_600_ of 2 with 1 mM IPTG for 6 to 10 hours or the time specified. Human CD20 cysteine residues 111 and 220 were mutated to serines by site directed mutagenesis to improve protein behavior during purification.

### Protein Purification

To determine protein location by detergent solubility, cells were lysed in buffer B (20 mM Tris, pH 7.5, 300 mM NaCl) by sonication and the membrane fraction was isolated by centrifugation. The membrane pellet was then re-suspended in buffer B and 1% Fos-Choline 12 (FC-12) and extracted overnight at 4°C. Samples were then centrifuged at 100,000×g for 1 hour and the supernatants collected. As necessary, the detergent soluble fraction was further purified using Ni-NTA Phynexus (San Jose, CA) pipette tips according to the manufacture's instructions.

For large-scale extraction, cells were re-suspended in 10 mL/g buffer A (20 mM Tris, pH 7.5, 5 mM EDTA) and centrifuged at 12,000×g for 30 min. The cell pellet was then re-suspended in buffer B (see above), lysed by cell disruption using a microfluidizer (Microfluidics Corp., Newton, MA) and centrifuged at 125,000×g for 1 hour. To extract the membrane protein from the cell membrane, the pellet was re-suspended in buffer B, FC-12 was added to 1% and the solution was stirred overnight at 4°C. The next day, the detergent insoluble fraction was pelleted by ultracentrifugation at 125,000×g for 1 hour. The supernatant was loaded onto a Ni-NTA Superflow (Qiagen Inc. Valencia, CA) column pre-equilibrated in buffer B containing 5 mM FC-12 (buffer C). The column was washed with 10 column volumes of 20 mM imidazole in buffer C and eluted with buffer C with 250 mM imidazole. All purification steps through column loading were performed at 4°C.

Eluent fractions containing CD20 were concentrated and loaded onto a Superdex 200 column (Amersham Biosciences, Piscataway, NJ) pre-equilibrated in buffer C. The his-tagged human CD20 was further purified over a 5 mL HiTrap HP Q (Amersham Biosciences, Piscataway, NJ) column prior to gel filtration. For LE-CD20, the LE leader was removed by thrombin before size exclusion chromatography.

For detergent exchange, samples were passed over a Superdex 200 column in 0.1% dodecyl maltoside, 150 mM NaCl, 20 mM HEPES, pH 7.2. Alternatively, samples were bound to a small Ni-NTA column, washed with buffer B and detergent and eluted in buffer B with detergent and 300 mM imidazole. These samples were then dialyzed against buffer B and detergent to remove imidazole.

### Density Gradient Centrifugation

A discontinuous sucrose gradient was generated by layering 1.75, 1.4 and 0.8 M sucrose solutions of Buffer D (150 mM NaCl and 20 mM HEPES, pH 7.2) in centrifuge tubes. *E. coli* membrane proteoliposome preparations were prepared by cell disruption in buffer D (10 mL/g) containing 1 mM EDTA. The insoluble fraction was isolated by centrifugation at 38,000×g for 1 hour. The supernatant was discarded and the pellet was re-suspended in Buffer D containing 0.25 M sucrose. This re-suspension was mixed with 1.9 M sucrose solution, resulting in final concentration of 1.75 M sucrose. 1 mL of this mixture was then placed at the bottom of a centrifuge tube and equal volumes of the 1.4 M and 0.8 M sucrose solutions were layered above. Samples were centrifuged for 1 hour at 100,000×g. Samples in 200 µL aliquots were removed from the top of the tube and analyzed by SDS-PAGE, transferred to nitrocellulose and probed with horseradish peroxidase conjugated anti-his antibody.

### ELISA Assays

96 well plates were coated overnight at 4°C with 100 µL of CD20 at 1 µg/mL in PBS with solubilizing detergent diluted to below its critical micelle concentration. Plates were then washed three times with PBS containing 0.05% Tween 20 (PBST) and blocked for 45 minutes at room temperature with 200 µL of PBST containing 0.5% BSA (blocking and assay buffer). Plates were washed again three times with PBST and probed with the primary antibody. 150 µL of rituximab at 60 µg/mL in assay buffer was added to the appropriate wells and three fold serial dilutions were performed in the subsequent wells by taking 50 µL from the first well and mixing with 100 µL of assay buffer in the next and subsequent wells to a final concentration of approximately 2 ng/mL. After 90 minutes incubation at room temperature, the plates were washed with PBST and bound rituximab was detected with 100 µL of horseradish peroxidase conjugated goat anti-human F(ab′)2 (Jackson ImmunoResearch Laboratories Inc, West Grove, PA) diluted 1∶2,000 in assay buffer, washed six times with PBST and developed with 100 µL/well of TMB Microwell Peroxidase Substrate System (KPL, Gaithersburg, MD) mixed according to the manufacturer's instructions. The reaction was halted by the addition of 100 µL/well of 1.0 M phosphoric acid and the absorbance was measured at 450 nm using a plate reader.

Reduced and alkylated CD20 samples were prepared by reduction with 10 mM DTT and alkylation by addition of 25 mM iodoacetamide. The reaction was halted by a further addition of 100 mM DTT. Following each step, the reaction was allowed to proceed for 30–60 minutes at room temperature at pH 8.0. EC_50_ values were determined by 4-parameter fit of the data.

### FACS Analysis

For preparation of spheroplasts, 5 OD_600_ mL of induced cells were recovered from expression media by centrifugation for 5 minutes at 5,000 rpm in a tabletop rotor (4,000×g). The supernatant was discarded and the pellet was re-suspended in 350 µL of ice-cold spheroplast preparation buffer A (50 mM Tris-HCl, pH 8.0, 25% sucrose, 100 µg/mL lysozyme, 67 µL/mL complete EDTA free protease inhibitor tablet in 2 mL deionized H_2_O) and the solution was diluted with 700 µL of ice-cold 1 mM EDTA. This mixture was allowed to incubate for 10 minutes at room temperature. 50 µL of 0.5 M MgCl_2_ was then added to stabilize the cell membrane and the mixture was incubated on ice for 10 minutes.

To block non-specific binding, the spheroplasts were pelleted at 5,000 rpm for 5 minutes in a tabletop centrifuge, gently re-suspended in 0.5 mL of ice-cold 10% fetal bovine serum in PBS and incubated on ice for 10 minutes. Spheroplasts were stained by addition of Alexa 488 conjugated anti-CD20 antibody at a concentration of 10 µg/mL followed by incubation at room temperature for 1 hour with mild agitation. Spheroplasts were pelleted as before and washed three times with 500 mL of PBS. Cells were analyzed on an EPIC-XL fluorescently activated cell sorter with the gating area adjusted for the size of the *E. coli* cells.

### ^35^S Pulse-Labeling

Cultures were induced for 30 minutes (14 hours for the late time point) with 1 mM ITPG at an OD_600_ of 2 and pulsed with ^35^S cysteine for 5 minutes. SDS was added to a final concentration of 2% to stop the labeling and then heated immediately at 95°C for 15 minutes to lyse the samples. The samples were then diluted with 2% FC-12 in PBS to bring down the SDS concentration to 0.2% so that they could be loaded onto a Ni-NTA spin column (Qiagen) and purified using a standard protocol provided by Qiagen. Eluates were separated by SDS-PAGE, transferred to nitrocellulose and exposed to a film.

## Supporting Information

Table S1 **Primary Protein Recovery.** Summary of protein yields after IMAC affinity purification from small-scale, 100 mL and large-scale, greater then 1 L expression.
(TIF)

Figure S1 **Restricted** ***E. coli*** **growth and small colony size formation following cell transformation with a multi-spanning membrane protein construct.** Basal protein expression from the *phoA* promoter is deleterious to cell growth. (a) vector control (b) *phoA*-RA1c expression construct uninduced (c) *tphac*-RA1c expression construct uninduced.
(TIF)

Figure S2 **Nucleotide sequence of the** ***phoA*** **,** ***phac*** **and** ***tphac*** **promoters.** (a) The *phoA* promoter showing the *pho* box and −10 sequences underlined (b) The dually regulated *phac* promoter showing the introduced *lac* operator underlined (c) The *tphac* promoter showing the addition of the λ t_o_ transcriptional terminator upstream of the *phac* promoter. *PhoA* −10 sequence and *Pho* Box (blue), *Lac* operator (red), Lambda transcription terminator (brown), ATG translation start (green).
(TIF)

Figure S3 **Membrane protein expression without a leader.** Comparison of the expression levels with the Uni and leaderless (Met) constructs for multi-spanning membrane proteins CD20, RA1c, EG-VEGFR1 and Patched 1. Arrows point to the monomer protein bands for the two GPCRs.
(TIF)

Figure S4 **N and C-terminal FLAG epitopes of LE-EG-VEGFR1 are accessible to anti-FLAG antibody.** Membrane proteoliposomes were prepared from *E. coli* expressing either N or C terminal FLAG tagged LE-EG-VEGFR1. Samples are: lane 1) pBR322 negative control; 2) LE-EG-VEGFR1, N-terminal FLAG; 3) LE-EG-VEGFR1, C-terminal FLAG; 4) pBR322 negative control; 5) LE-EG-VEGFR1, N-terminal FLAG; 6) LE-EG-VEGFR1, C-terminal FLAG. Samples for lanes one, two and three were treated with 1% Triton X-100 prior to incubation with anti-FLAG antibody. Samples for lanes four, five and six were treated with antibody in the absence of detergent.
(TIF)

Figure S5 **Extraction of LE-CD20 from the cell membrane.** Samples of *E. coli* membrane with expressed LE-CD20 were treated with a ratio of detergents from 1% FC-12 to 1% DDM. Lane 1) 1% FC-12; 2) 0.75∶0.25; 3) 0.5∶0.5; 4) 0.25∶0.75; 5) 1.0% DDM. Membrane samples were extracted with detergent over night and CD20 was detected using an anti-His HRP conjugated antibody.
(TIF)

Figure S6 **Representative gels of membrane proteins following large-scale purification over immobilized nickel column.** Samples were detected by coomassie staining following separation on 4 to 20% SDS-PAGE. Samples are: lane 1) LE-CD20; 2) Molecular weight marker; 3) LE-EG-VEGF-R1; 4) LE-RA1c; 5) Molecular weight markers. Each sample lane contains 15 µg of protein. Molecular weights of the protein standards are shown on side of the figure.
(TIF)

Figure S7 **LE-CD20 is expressed at high levels in** ***E. coli*** **.** Total cellular level of LE-CD20 was determined by comparison to a standard curve generated with the purified protein. Based on the average OD_600_ of 3.0 for the LE-CD20 culture, total expression is 41 milligrams per liter of culture. Representative data from two independent measurements is shown. *Lane quantitation was determined using Licor-700.
(TIF)

Methods S1 **Methods for quantitation of LE-CD20 expression levels in** ***E. Coli*** **.**
(DOC)
