# Supplementary figures and images for: Translation Levels Control Multi-Spanning Membrane Protein Expression
Source: PLoS One. 2012 Apr 26;7(4):e35844. doi: 10.1371/journal.pone.0035844 (PMC3338534; doi:10.1371/journal.pone.0035844)

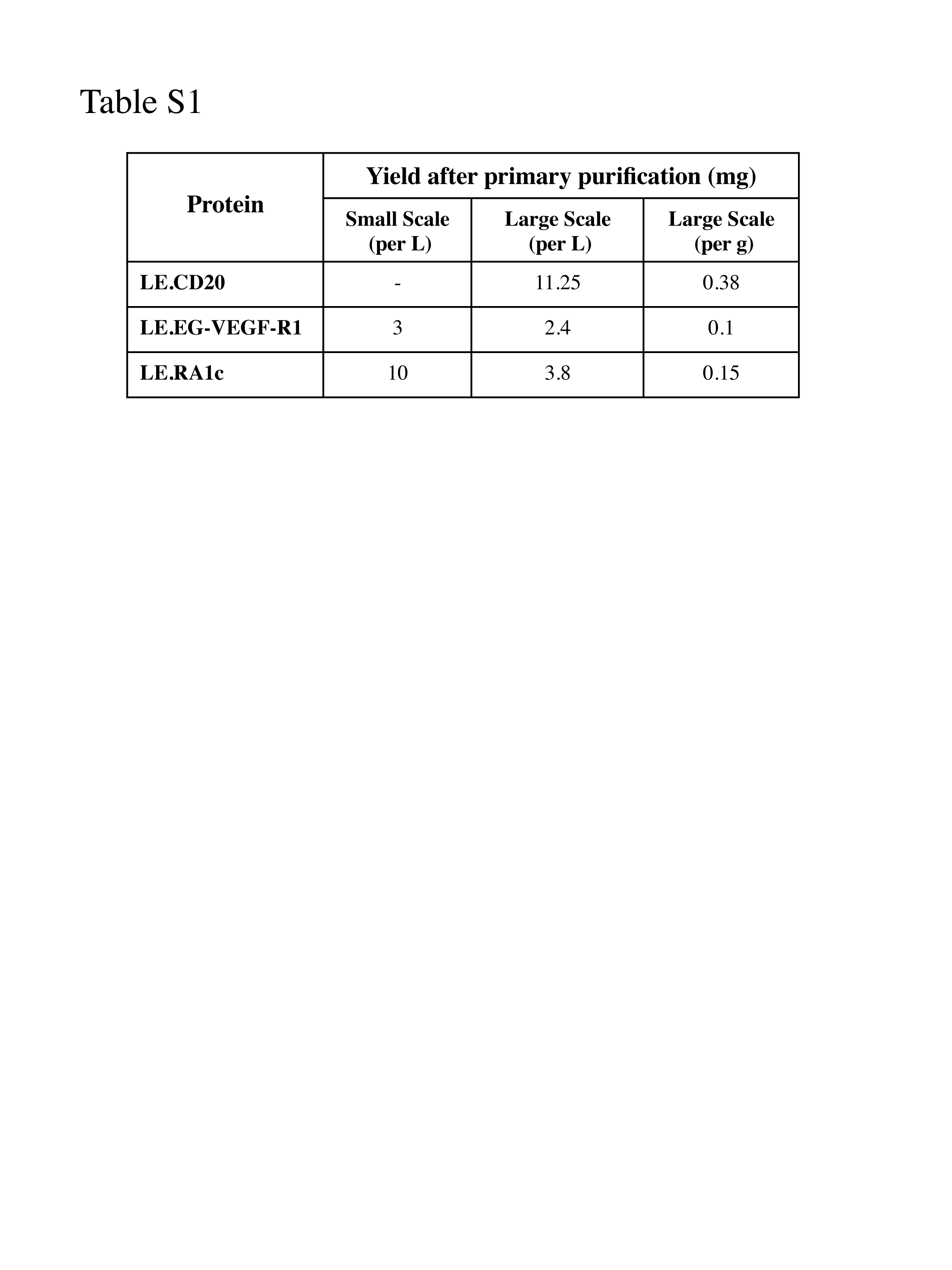

Supplement: Table S1 — Primary Protein Recovery. Summary of protein yields after IMAC affinity purification from small-scale, 100 mL and large-scale, greater then 1 L expression. (TIF) [file pone.0035844.s001.tif]

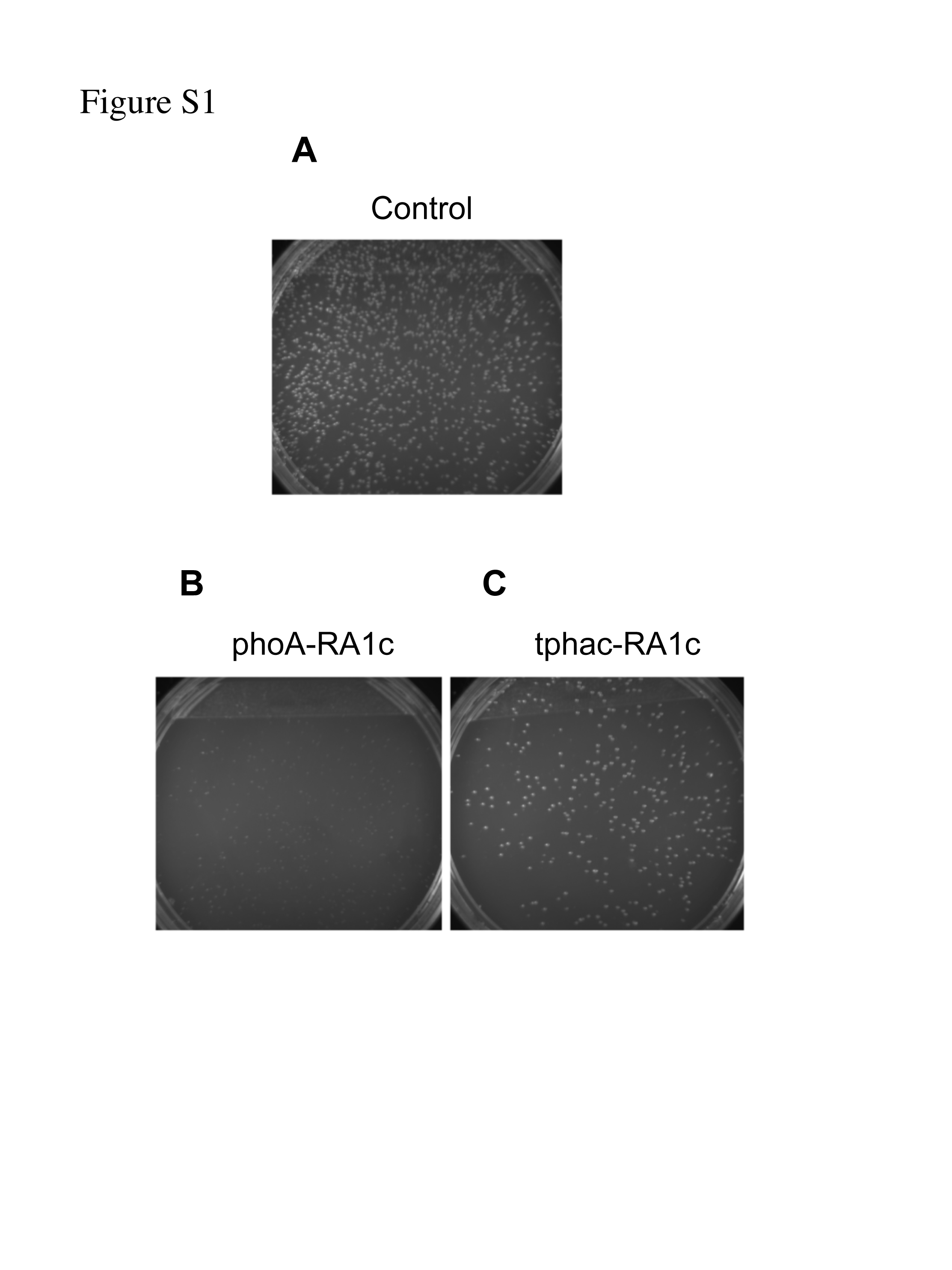

Supplement: Figure S1 — Restricted E. coli growth and small colony size formation following cell transformation with a multi-spanning membrane protein construct. Basal protein expression from the phoA promoter is deleterious to cell growth. (a) vector control (b) phoA-RA1c expression construct uninduced (c) tphac-RA1c expression construct uninduced. (TIF) [file pone.0035844.s002.tif]

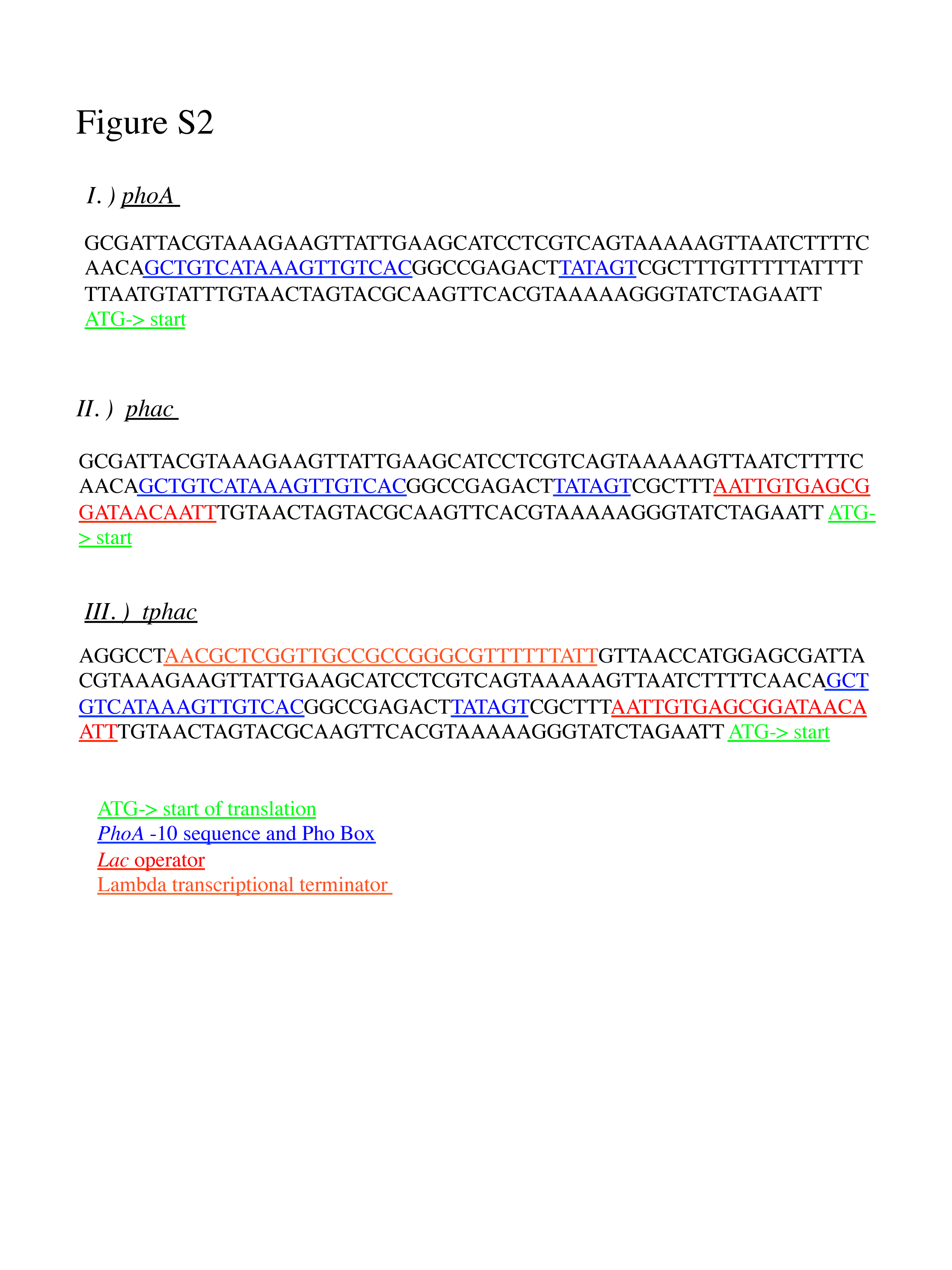

Supplement: Figure S2 — Nucleotide sequence of the phoA , phac and tphac promoters. (a) The phoA promoter showing the pho box and −10 sequences underlined (b) The dually regulated phac promoter showing the introduced lac operator underlined (c) The tphac promoter showing the addition of the λ to transcriptional terminator upstream of the phac promoter. PhoA −10 sequence and Pho Box (blue), Lac operator (red), Lambda transcription terminator (brown), ATG translation start (green). (TIF) [file pone.0035844.s003.tif]

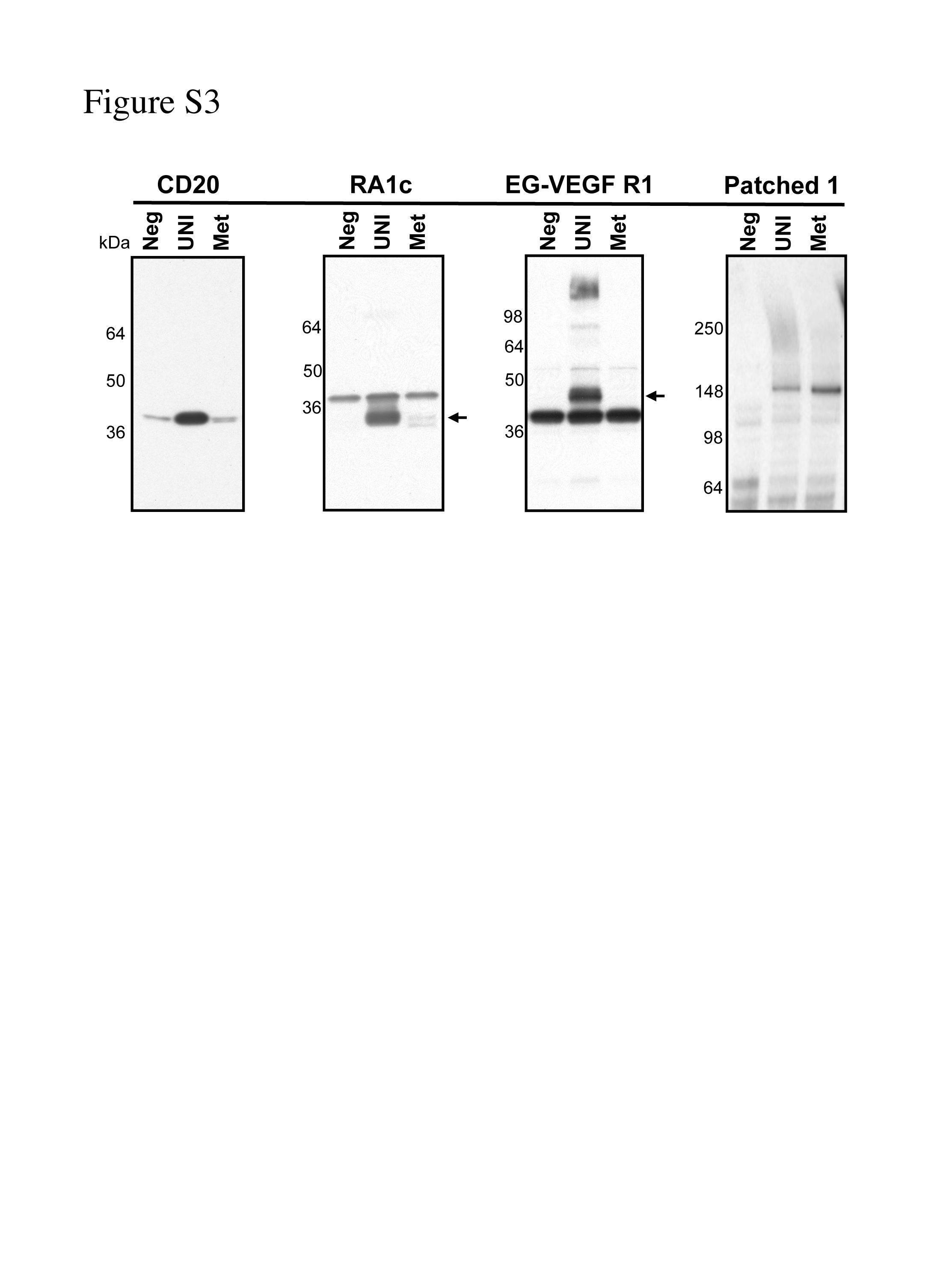

Supplement: Figure S3 — Membrane protein expression without a leader. Comparison of the expression levels with the Uni and leaderless (Met) constructs for multi-spanning membrane proteins CD20, RA1c, EG-VEGFR1 and Patched 1. Arrows point to the monomer protein bands for the two GPCRs. (TIF) [file pone.0035844.s004.tif]

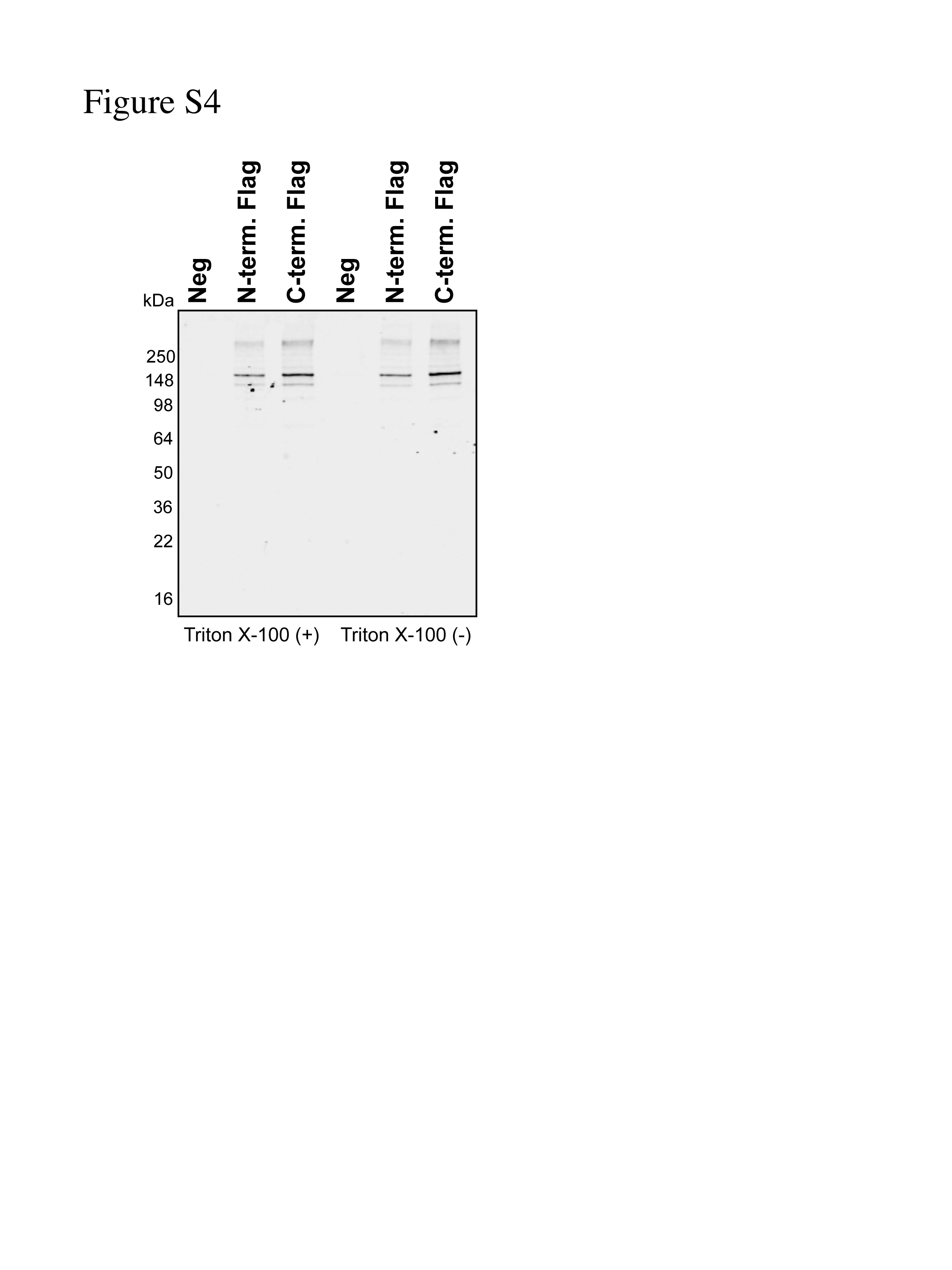

Supplement: Figure S4 — N and C-terminal FLAG epitopes of LE-EG-VEGFR1 are accessible to anti-FLAG antibody. Membrane proteoliposomes were prepared from E. coli expressing either N or C terminal FLAG tagged LE-EG-VEGFR1. Samples are: lane 1) pBR322 negative control; 2) LE-EG-VEGFR1, N-terminal FLAG; 3) LE-EG-VEGFR1, C-terminal FLAG; 4) pBR322 negative control; 5) LE-EG-VEGFR1, N-terminal FLAG; 6) LE-EG-VEGFR1, C-terminal FLAG. Samples for lanes one, two and three were treated with 1% Triton X-100 prior to incubation with anti-FLAG antibody. Samples for lanes four, five and six were treated with antibody in the absence of detergent. (TIF) [file pone.0035844.s005.tif]

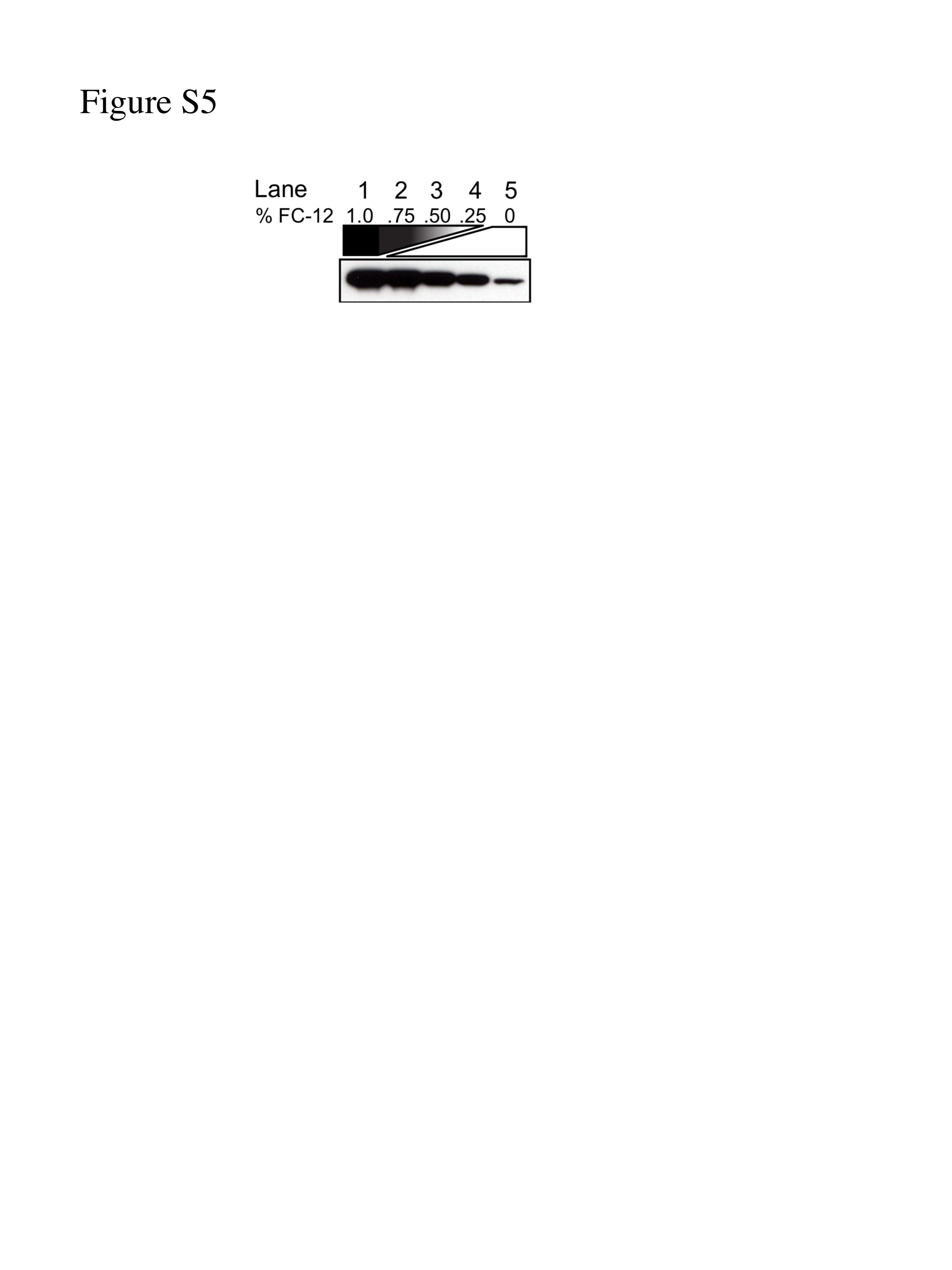

Supplement: Figure S5 — Extraction of LE-CD20 from the cell membrane. Samples of E. coli membrane with expressed LE-CD20 were treated with a ratio of detergents from 1% FC-12 to 1% DDM. Lane 1) 1% FC-12; 2) 0.75∶0.25; 3) 0.5∶0.5; 4) 0.25∶0.75; 5) 1.0% DDM. Membrane samples were extracted with detergent over night and CD20 was detected using an anti-His HRP conjugated antibody. (TIF) [file pone.0035844.s006.tif]

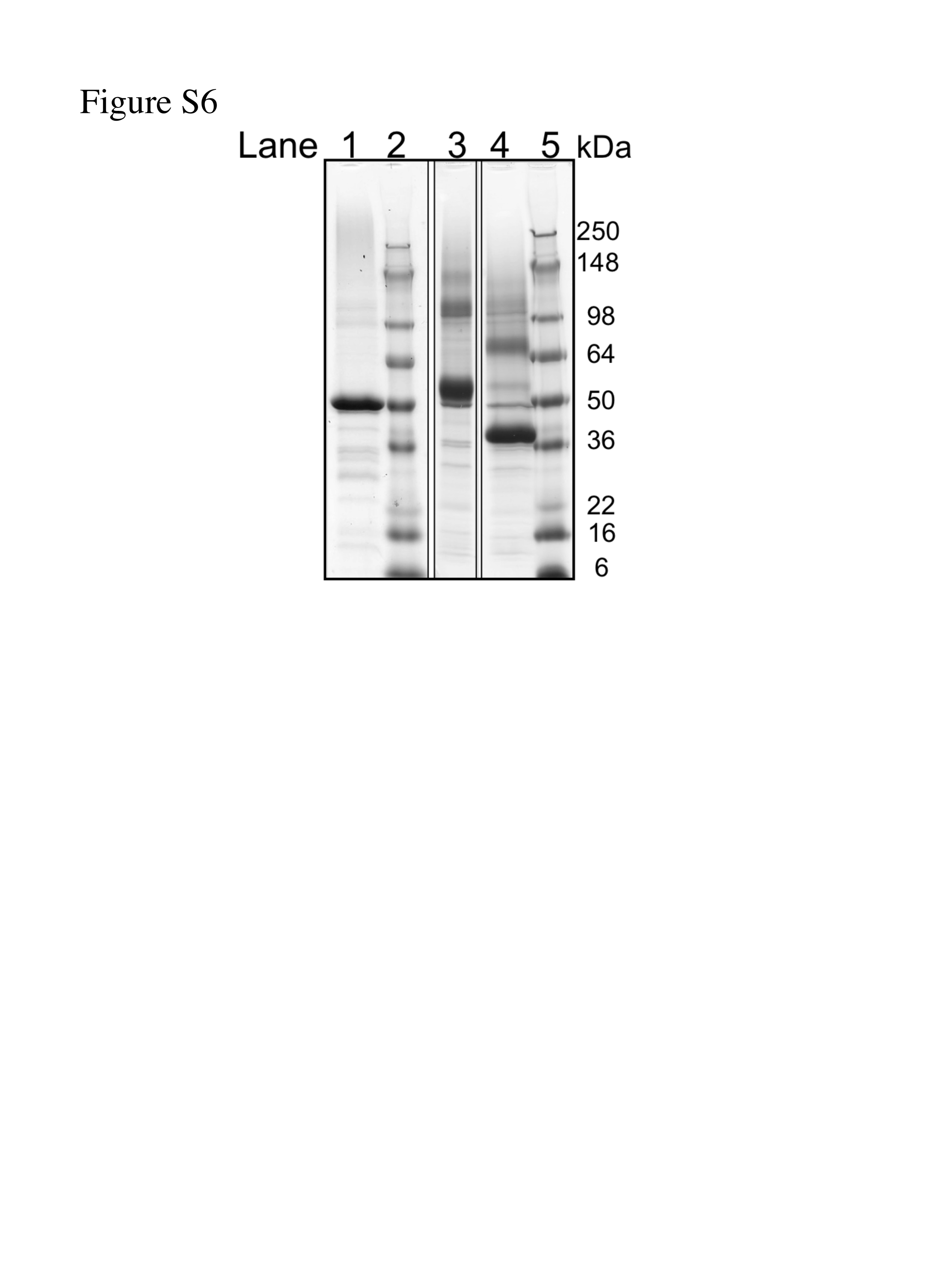

Supplement: Figure S6 — Representative gels of membrane proteins following large-scale purification over immobilized nickel column. Samples were detected by coomassie staining following separation on 4 to 20% SDS-PAGE. Samples are: lane 1) LE-CD20; 2) Molecular weight marker; 3) LE-EG-VEGF-R1; 4) LE-RA1c; 5) Molecular weight markers. Each sample lane contains 15 µg of protein. Molecular weights of the protein standards are shown on side of the figure. (TIF) [file pone.0035844.s007.tif]

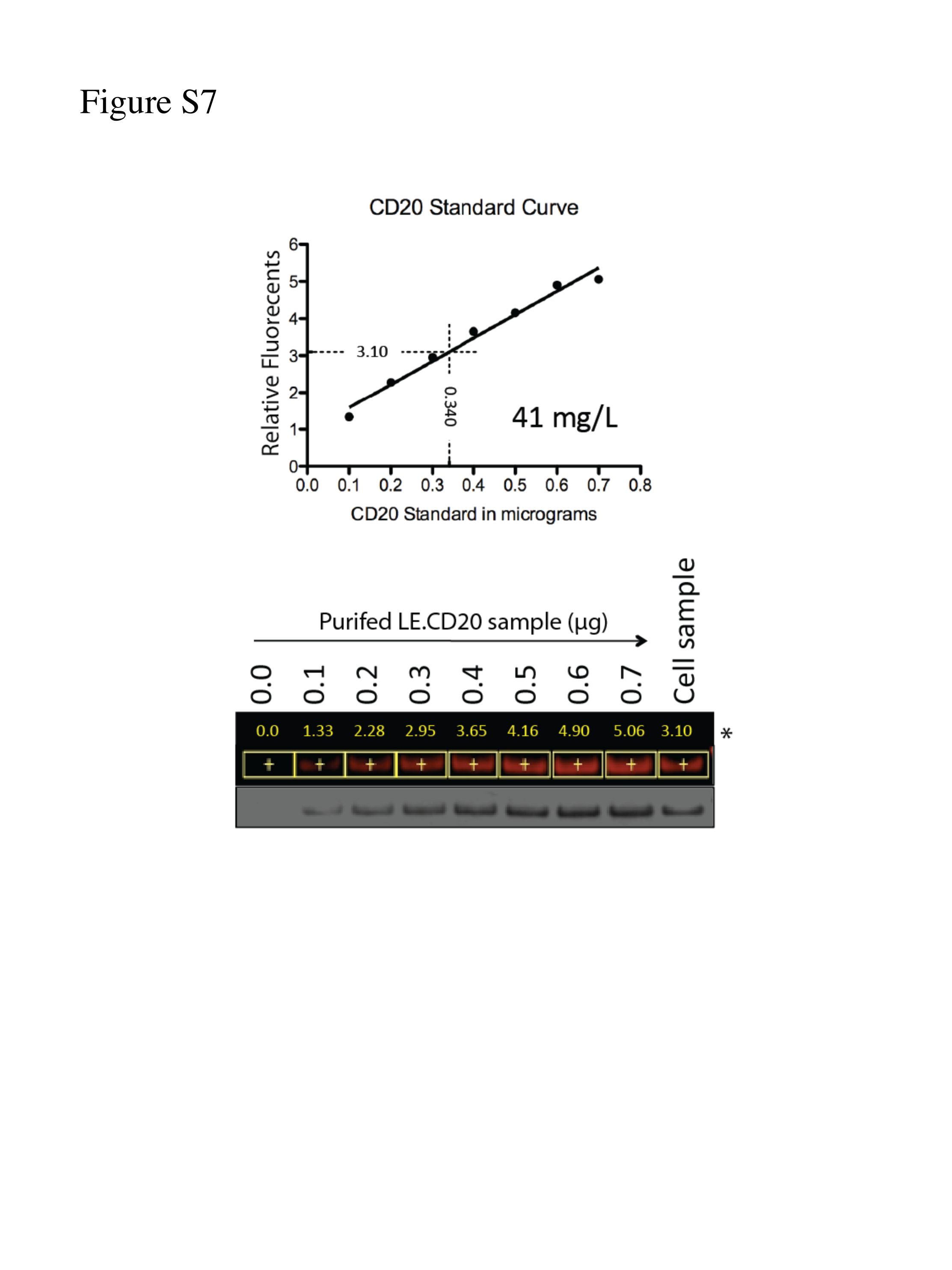

Supplement: Figure S7 — LE-CD20 is expressed at high levels in E. coli . Total cellular level of LE-CD20 was determined by comparison to a standard curve generated with the purified protein. Based on the average OD600 of 3.0 for the LE-CD20 culture, total expression is 41 milligrams per liter of culture. Representative data from two independent measurements is shown. *Lane quantitation was determined using Licor-700. (TIF) [file pone.0035844.s008.tif]
