# Supplementary material for: Translation Levels Control Multi-Spanning Membrane Protein Expression
Source: PLoS One. 2012 Apr 26;7(4):e35844. doi: 10.1371/journal.pone.0035844 (PMC3338534; doi:10.1371/journal.pone.0035844)
Supplement: Methods S1 — Methods for quantitation of LE-CD20 expression levels in E. Coli . (DOC) [file pone.0035844.s009.doc]

**Supporting Methods**

**Methods for quantitation of LE-CD20 expression levels in *E. Coli*.** LE-CD20 standards were prepared from purified LE-CD20 with a known concentration (0.8 mg/mL). Standards from 0.1 to 0.7 µg were compared to 0.025 OD_600_ of LE-CD20 whole cell lysate of unknown concentration. All samples were loaded onto a NuPAGE Bis-Tris gel and western blotted onto a PVDF membrane. Membrane was blocked with gelatin for one hour and incubated with Mouse anti-His antibody from Roche for overnight. Membrane was washed and incubated with the secondary antibody, anti-Mouse-Licor700 for 20 minutes. The sample readings were obtained with the LI-COR Odyssey Infrared Imaging System. The unknown LE-CD20 sample amount was determined using the PRISM standard curve analysis.
